# Excitation/Inhibition Modulators in Autism Spectrum Disorder: Current Clinical Research

**DOI:** 10.3389/fnins.2021.753274

**Published:** 2021-11-30

**Authors:** Roberto Canitano, Roberto Palumbi

**Affiliations:** ^1^Division of Child and Adolescent Neuropsychiatry, University Hospital of Siena, Siena, Italy; ^2^Division of Child and Adolescent Neuropsychiatry, Basic Medical Sciences, Neuroscience and Sense Organs Department, University Hospital of Bari, Bari, Italy

**Keywords:** children and adolescents, autism spectrum disorders, excitation/inhibition imbalance, experimental treatments, excitation/inhibition modulators

## Abstract

Autism spectrum disorder (ASD) is a group of neurodevelopmental disorders characterized by social and communication abnormalities. Heterogeneity in the expression and severity of the core and associated symptoms poses difficulties in classification and the overall clinical approach. Synaptic abnormalities have been observed in preclinical ASD models. They are thought to play a major role in clinical functional abnormalities and might be modified by targeted interventions. An imbalance in excitatory to inhibitory neurotransmission (E/I imbalance), through altered glutamatergic and GABAergic neurotransmission, respectively, is thought to be implicated in the pathogenesis of ASD. Glutamatergic and GABAergic agents have been tested in clinical trials with encouraging results as to efficacy and tolerability. Further studies are needed to confirm the role of E/I modulators in the treatment of ASD and on the safety and efficacy of the current agents.

## Introduction

### Characterizing Autism Spectrum Disorder

Autism spectrum disorder (ASD) is a group of complex neurodevelopmental disorders characterized by the presence of multiple and persistent deficits in social communication and social interaction associated with restricted interests and stereotyped and repetitive behaviors (American Psychiatric Association, 2013; Grzadzinski et al., 2013). The prevalence of ASD is on the rise, as documented in recent surveys (Baio et al., 2018). There are more males than females with ASD (a 4:1 ratio), and there are relevant clinical differences between the sexes (Tillmann et al., 2018).

The etiology of ASD is still poorly understood, but it appears highly genetically heritable. Indeed, there are identified genetic mutations—although not fully penetrant—in approximately 15% of the cases. Non-specific environmental risks factors, such as parental age, fetal exposure to alcohol or valproate, and premature birth/low birth weight, have also been identified (Murdoch and State, 2013; De Rubeis and Buxbaum, 2015; Sestan and State, 2018; Han et al., 2021a,b). The most frequent comorbidities and associated symptoms are learning/intellectual disabilities, sleep disturbances, epilepsy, attention deficit with hyperactivity, irritability (including self-injuries behavior, and temper tantrums), anxiety, and depression. Frequently used medications include methylphenidate and atomoxetine for attention disorders, melatonin for sleep disorders, and selective serotonin reuptake inhibitors (SSRIs) for anxious-depressive symptoms. Second-generation antipsychotics, namely risperidone, and aripiprazole have demonstrated clinical efficacy in behavioral disorders of ASD, and the United States Food and Drug Administration (FDA) has approved them for this use (Accordino et al., 2016). The presence and the severity of these symptoms are very heterogeneous among patients. Usual care includes psychosocial interventions as well as educational measures, patient and family support, and behavioral therapies such as applied behavioral analysis.

The considerable heterogeneity in the expression and severity of the core and associated symptoms have been an obstacle toward understanding ASD. Variability in the social domain ranges from a near absence of interest in interacting with others to more subtle difficulties managing complex social interactions that require an understanding of other people’s goals and intentions and social context cues (American Psychiatric Association, 2013; Grzadzinski et al., 2013). Similarly, repetitive behaviors range from simple motor stereotypies and/or a preference for sameness to much more complex and elaborate rituals accompanied by emotional dysregulation or tantrums when these rituals are interrupted. Some individuals with ASD lack basic speech abilities, while others can have language deficits that are mild and limited to language pragmatics. While a majority of individuals with ASD exhibit some level of intellectual impairment, intelligence quotients vary from the severe and profoundly impaired to above-average (Frye, 2018).

### Models of Excitatory/Inhibitory Imbalance in Autism Spectrum Disorder

A theory of excitatory/inhibitory imbalance in ASD was proposed by Rubenstein and Merzenich (2003). This neurobiological model is based on the theory that autism and related disorders might reflect an increase in the ratio between excitation and inhibition leading to hyper-excitability of cortical circuits. The theory was attractive because it provided a potential explanation for the frequent observation of reduced GABAergic signaling in ASD (Cellot and Cherubini, 2014; Brondino et al., 2016). In addition, since inhibition was known or believed to contribute to sharpening the selectivity of excitatory responses in many brain areas, the loss of inhibition could lead to enhanced “noise” and imprecision in learning and cognition (Rubenstein and Merzenich, 2003).

Since this initial formulation, however, other studies have suggested a nearly opposite hypothesis; namely, that at least some ASDs might be characterized by a reduction in the ratio between excitation and inhibition (Vignoli et al., 2010; Spooren et al., 2012). An excitatory/inhibitory (E/I) neurotransmission imbalance in cortical neurons, using dysfunctional glutamatergic and GABAergic neurotransmission, has been reported in multiple studies, and altered glutamine levels and/or abnormal GABA/creatine levels in brain tissue or plasma are the relevant findings (Vignoli et al., 2010; Carlson, 2012; Spooren et al., 2012; Gao and Penzes, 2015; Dickinson et al., 2016; Lee et al., 2017; Al-Otaish et al., 2018; Goel and Portera-Cailliau, 2019; Port et al., 2019; Bruining et al., 2020; Culotta and Penzes, 2020). Moreover, a recent study showed that high glutamate/glutamine levels have also been associated with altered functional connectivity in key brain regions for ASD, such as the dorsal anterior cingulate cortex, and insular, limbic, and parietal regions (Siegel-Ramsay et al., 2021).

An E/I imbalance might be the consequence of an increased glutamatergic activity alongside with a decrease in the GABAergic signaling. Previous studies found that genes involved in the regulation of the neurogenesis or the synaptogenesis may be linked to the E/I imbalance (Vignoli et al., 2010). In neurodevelopmental disorders, an E/I imbalance could arise directly through alterations in genes coding for glutamatergic receptors or synaptic proteins (Lee et al., 2017; Culotta and Penzes, 2020). The synapse organizers, neurexins and their binding neuroligins, are implicated in the formation and maintenance of excitatory and inhibitory synapses. Heterozygous deletions eliminating exons of the neurexin-1α gene in patients with ASD have been detected, and the functional significance of this recurrent deletion is still unclear (Frye, 2018). However, the role of neurexins/neuroligins in the pathogenesis of neurodevelopmental disorders has been demonstrated (Dickinson et al., 2016; Al-Otaish et al., 2018; Wang et al., 2018; Goel and Portera-Cailliau, 2019; Port et al., 2019; Siegel-Ramsay et al., 2021). The final effect of changes in glutamatergic and GABAergic systems in ASD may be an overall increase in the ratio of excitation to inhibition (E/I) (Howell and Smith, 2019). The E-I ratio in neocortical structures is determined by pyramidal glutamatergic neurons and inhibitory GABAergic parvalbumin (PV)-positive interneurons that are modulated by minicolumns, i.e., groups of functionally autonomous neurons whose afferent and efferent connections influence the functioning of microcircuits), which are abnormal in ASD (Nelson and Valakh, 2015; Uzunova et al., 2016). There are several candidate mechanisms for glutamatergic hyperexcitability. Neuroligins (NL1-4) and neurexins (Nrxns 1–3) have been linked with ASD *via* point mutations and truncations, as well as chromosomal rearrangements that have been identified in the region of interest (Snijders et al., 2013; Port et al., 2014; Dickinson et al., 2016). SHANK1, SHANK2, and SHANK3 are scaffolding proteins that influence the postsynaptic density of glutamatergic synapses and are of primary importance in ASD. SHANK3 is reported to be involved in Phelan-McDermid syndrome (PMS) a form of ASD associated with moderate to severe intellectual disability (ID). Regarding SHANK2 and SHANK1, they were found altered in ASD associated with mild ID as well as in high functioning individuals (Zikopoulos and Barbas, 2013; Cochran et al., 2015; Howell and Smith, 2019; Bruining et al., 2020; Culotta and Penzes, 2020; Siegel-Ramsay et al., 2021). As to the mechanisms of GABAergic inhibitory dysfunction, the link with core ASD symptoms in humans is still under investigation. The deficit in binocular rivalry, a visual function that is thought to rely on the balance of excitation/inhibition in the visual cortex, has been observed in ASD individuals (Dunn and Jones, 2020). The link between GABA and binocular rivalry dynamics was found specifically absent in ASD pointing to an insufficient GABA inhibitory function (Dunn and Jones, 2020).

Based on these findings, research efforts have been directed to identify agents that have the potential to reverse this abnormal signaling, namely, excitation to inhibition (E/I) imbalance from preclinical models to clinical trials.

## Modulators of E/I Imbalance in Autism Spectrum Disorder

Modulators of E/I imbalance are a group of agents that are used to restore the balance of excitation and inhibition in brain cortical regions that are dysfunctional in ASD patients. These agents include compounds that target metabotropic glutamate receptors (mGluR, e.g., mGluR5 antagonists), NMDA receptors (e.g., the NMDA receptor antagonist memantine), or AMPA receptors (with receptor potentiating drugs such as ampakines). The search of articles was performed on PubMed online database, published until May 2021 on peer-reviewed journals. Studies identified had to meet predetermined inclusion criteria: (1) randomized clinical trial; (2) written only in English; (3) patients diagnosed with ASD; (4) E/I imbalance modulators in ASD; (5) full-text availability. Only few randomized clinical trial placebo-controlled studies have been performed to date and will be dealt with in the current article (Table 1).

**TABLE 1:** Randomized control trials (RCT) with excitatory/inhibitory (E/I) modulators in ASD.

	RCT	Age of participants and number (N)	Medication dosage mg/day	Effect size (d)	Outcome measures	Main findings
*Arbaclofen*						
Veenstra-VanderWeele et al., 2017	12 week randomized placebo-controlled	5–21 years *N* = 150	5–10 mg	NS	ABC, CGI	No differences between placebo and treatment. CGI scores improvement
*Bumetanide*						
Lemonnier et al., 2012 Lemonnier et al., 2017	12 week randomized double-blind 12 week randomized, double-blind, placebo-controlled (phase 2B trial)	3–11 years *N* = 60 2–17 years *N* = 88	0.5 mg 0.5, 1.0- 2.0 mg twice daily	NS	CARS, CGI, ADOS CARS, CGI, SRS	No differences between placebo and treatment CGI scores improvement CARS, CGI, SRS scores significantly improved
*Memantine*						
Ghaleiha et al., 2013 Aman et al., 2017	10 week, double-blind (1) 12 week parallel-group, flexible-dose (2) 48 week open-label extension	4–12 years *N* = 40 6–12 years *N* = 121 *N* = 102	5 mg daily Weight 60 kg, max 15 mg/day; 40–59 kg, max 9 mg/day; 20–39 kg, max 6 mg/day; <20 kg, max 3 mg/day.	NS NS	ABC-C SRS	Significant improvement in irritability, stereotyped behaviors and hyperactivity in memantine treated group No differences between placebo and treatment at the end of week 12 Trend of improvement at the end of week 48 extension
*N-acetyl cysteine (NAC)*						
Hardan et al., 2012 Ghanizadeh and Moghimi-Sarani, 2013 Nikoo et al., 2015 Dean et al., 2017 Wink et al., 2016	12 week, double-blind, placebo-controlled 8 week double-blind placebo-controlled 10 week randomized, double-blind, placebo-controlled 6 months double-blind, placebo-controlled 12-week randomized, double-blind, placebo-controlled	3.2–10.7 years *N* = 33 3.5–16 years *N* = 40 4–12 years *N* = 40 3.1–9.9 years *N* = 98 4–12 years *N* = 31	900 mg daily for 4 weeks, then 900 mg twice-daily for 4 weeks and 900 mg three-times-daily for 4 weeks 1,200 mg daily Risperidone 1- 2 mg/d; NAC 600–900 mg/day 500 mg/day Target dose of NAC: 60 mg/kg/day	0.96 NS 0.99 NS 0.7 NS 0.75 NS NS NS	ABC-I ABC-Stereotypy subscale SRS social SRS ABC-I ABC-SW ABC–I ABC-SW ABC-C irritability subscale SRS, CCC2, RBS-R CGI-I	Significant improvements on ABC-Irritability subscale Significant improvements in the group treated with NAC + risperidone vs. placebo + risperidone treated group Significant reduction in irritability and hyperactivity scales in the group treated with NAC + risperidone vs. placebo + risperidone treated group No significant differences between placebo and NAC treated group on any of the outcome measure No significant improvement on social impairment between NAC and placebo groups

*ADOS, Autism Diagnostic Observation Scale; ABC, Aberrant Behavior Checklist; ABC-I, ABC-Irritability subscale; ABC-SW, ABC-Social Withdrawal subscale; ABC-C, ABC–Communication Scale; CARS, Childhood Autism Rating Scale; CGI-I, CGI-Improvement; CGI-S, CGI-Severity Scale; CGI-TI, CGI Therapeutic Index; CCC-2, Children’s Communication Checklist; RBS-R, Repetitive Behaviors Scale-Revised; SRS, Social Responsiveness Scale; NS, not significant.*

### Glutamatergic and GABAergic Modulators in Autism Spectrum Disorder

Dysfunctional glutamatergic transmission occurs through different pathways of altered excitatory transmission: downregulation of AMPA receptors, abnormalities in NMDA-receptor-mediated plasticity, and altered mGluR signal transduction. Research has implicated glutamate hyperactivation and GABAergic system hypofunction in the pathogenesis of ASD. In neurons and glia, glutamate is involved in intracellular transduction mechanisms at NMDA and mGlu receptors (Uzunova et al., 2014). AMPA receptors are thought to be implicated in behavioral disorders associated with the core symptoms in children and adolescents with ASD (Lipton, 2006; Uzunova et al., 2014).

#### NMDA Receptor Antagonist: Memantine Treatment Studies

NMDA receptor antagonists, mainly memantine, have been employed in clinical trials for children and adolescents with ASD. Memantine is an uncompetitive, low-affinity glutamate NMDA receptor antagonist used for this purpose (Lipton, 2006; Wei et al., 2012). Owley et al. (2006) enrolled 14 subjects, aged 3–12 years, in an 8-week open-label study of memantine for ASD. The patients received up to 20 mg/day memantine. There were significant improvements in memory, irritability, lethargy, stereotypies, hyperactivity, and inappropriate speech (Owley et al., 2006). A retrospective study by Erickson et al. (2007) evaluated the use of memantine in 18 ASD patients (6–19 years old). The maximum dose of memantine was 20 mg/day. The outcome measures included the Clinical Global Impression (CGI)–Severity and CGI–Improvement subscales and Aberrant Behavior Checklist (ABC) data. They reported a significant reduction in hyperactivity. Chez et al. (2007) conducted an open-label study of 151 patients with autism or “Pervasive Developmental Disorder–Not Otherwise Specified” with an age range of 2.5–26.3 years. The patients received a maximum dosage of 30 mg/day of memantine. There was a clinical improvement in 70% of patients, including language, social behavior, and stereotypes as reported by the CGI scale. There was worsened irritability, hyperactivity, and manic-type behaviors in 18/150 [11.84%] of patients. A double-blind multisite study was carried out to assess the safety, tolerability, and efficacy of memantine [once-daily extended-release (ER)] in children with autism in a randomized, placebo-controlled, 12-week trial and a 48-week open-label extension (Aman et al., 2017). A group of 121 children (6–12 years) with autistic disorder (based on the DSM-IV-TR) were randomized to placebo or memantine ER for 12 weeks; 104 children entered the subsequent extension trial. The maximum memantine doses were determined by body weight; they ranged from 3 to 15 mg/day. There was no significant between-group difference on the primary efficacy outcome of caregiver/parent ratings on the Social Responsiveness Scale (SRS). This trial did not demonstrate the clinical efficacy of memantine ER in autism; however, the tolerability and safety were good.

Another double-blind, placebo-controlled study explored memantine as add-on therapy in children with ASD receiving risperidone (Ghaleiha et al., 2013). The addition of memantine significantly reduced ABC subscale scores for irritability, hyperactivity, and stereotypic behavior compared to placebo. These findings suggest that memantine can be an effective adjunctive treatment for behavioral problems in children with ASD.

#### GABAergic Modulator: STX209 Treatment Studies

Multiple studies have suggested that GABAergic neurons and circuits may be altered in ASD (Chao et al., 2010; Port et al., 2017). GABA neurotransmission regulates several important developmental processes, including cell proliferation, differentiation, synapse maturation, and cell death. Dysfunctional GABAergic signaling early in development that leads to a severe E/I imbalance in neuronal circuits has been observed in ASD in studies conducted with different methods (Oblak et al., 2010; Ghanizadeh and Moghimi-Sarani, 2013; Gaetz et al., 2014). GABAergic modulators have been tested in ASD. An open-label trial evaluated the safety, tolerability, and efficacy of STX209, a form of arbaclofen, in ASD individuals (Erickson et al., 2014). There were improvements in several outcome measures, and Following these positive preliminary findings, a 12-week randomized controlled trial of STX209 was conducted (Veenstra-VanderWeele et al., 2017). STX209 was more efficacious compared to placebo, even if the results did not reach statistical significance.

#### GABAergic Modulator: Bumetanide Treatment Studies

The persistence or development of excitatory GABA neuronal signaling has been found in ASD individuals, and the E/I imbalance is hypothesized to be related to defect in GABAergic signaling (Bruining et al., 2015; Ben-Ari, 2017). Bumetanide is a loop diuretic and it could reduce intracellular chloride and thus reverse the aberrant excitatory action of GABA into inhibitory action (Lemonnier and Ben-Ari, 2010). The first use of bumetanide in ASD was a pilot study conducted in five children with ASD (3–11) years old. Bumetanide in ASD was tested in preliminary studies (Lemonnier and Ben-Ari, 2010; Lemonnier et al., 2012; Bruining et al., 2015; Hadjikhani et al., 2015), followed by a proof of concept study (Hadjikhani et al., 2015).

Lemonnier et al. (2012) conducted a randomized, double-blind, placebo-controlled trial evaluating the efficacy of bumetanide treatment in 60 French children with ASD. The group treated with the bumetanide showed a statistically significant improvement in the mean total of the Childhood Autism Rating Scale (CARS) (Schopler et al., 2009) score (5.6 ± 4 compared with placebo 1.8 ± 5.1; *P* = 0.0044) after 3 months. Moreover, the treatment ameliorated also the Autism Diagnostic Observation Schedule (ADOS) (Lord et al., 2000) scores, even if the results were not statistically significant. Du et al. (2015) conducted a randomized trial in 60 Chinese children with ASD (2.5–6.5 years old), with a mean of 4.5 years, treatment with bumetanide combined with ABA training provided a better outcome than ABA training alone.

Lemonnier et al. (2017) carried out another double-blind, randomized controlled, multicenter dose-ranging study. The authors identified responders of all ages and ASD severity groups, according to CARS scores. However, it was unclear whether patients with more severe ASD symptoms have a greater response to bumetanide. Bumetanide is therefore promising in treating ASD, including core symptoms, and await for further studies.

### Brain Glutamate Modulator: *N*-Acetylcysteine Treatment Studies

Glutathione and *N*-Acetylcysteine (NAC) are antioxidants that minimize oxidative stress and the downstream negative effects thought to be associated with oxidative stress. Based on magnetic resonance imaging studies, NAC modulates brain glutamate and can thus potentially modulate the E/I balance (Deepmala et al., 2015; Minarini et al., 2017).

In Hardan’s et al. 12-week double-blind, placebo-controlled randomized study of children with ASD (*n* = 33), 14 subjects in the NAC group and 15 in the placebo group completed the trial. Oral NAC was well tolerated with limited side effects (Hardan et al., 2012). Wink et al. (2016) reported no statistically significant difference between the NAC and placebo groups on the CGI-Improvement (*P* > 0.69), but glutathione was significantly higher in the NAC group (*P* < 0.05). NAC treatment was well tolerated but had no significant impact on social impairment in youth with ASD (Wink et al., 2016). Dean et al. (2017) examined a total of 102 children; 98 (79 males and 19 females; age range: 3.1–9.9 years) formed the study group. There were no differences between the NAC and placebo groups on any of the outcome measures for either primary or secondary endpoints. In a double-blind, placebo-controlled add-on study to risperidone (*n* = 40), 600 mg of NAC twice a day significantly decreased ABC-Irritability but did not change ABC-Social Withdrawal (Nikoo et al., 2015).

The limitations of the reviewed studies include the potential lack of sensitivity of the scales used to assess real improvement and behavioral changes, the absence of biological markers that would allow correlation of any clinical improvement with biological changes, and the heterogeneity of ASD symptoms. Additional randomized double-blind, placebo-controlled trials with the presented E/I modulators are required to draw firm conclusions.

## Conclusion

The currently available agents that modulate E/I imbalance herein discussed act upon selective symptoms of ASD, namely irritability, and some of them (e.g., bumetanide) hold promise to modify the abnormalities in social and communication domains as well. Differences in response to the tested agents have been identified across development in subjects with ASD, and the same has been observed about tolerability. Thus, a careful distinction in age groups should be detailed when setting up study designs and evaluating treatment response. The response to placebo and the level of baseline symptomatology should be considered in the design and interpretation of future trials in ASD. It is very important to stratify the patients according to the symptom level—e.g., social avoidance and lack of interest in reciprocity may vary—so it is urgently necessary to specify it and consider the distinctly associated biomarkers.

Target symptom domains of ASD have been identified and basic mechanisms that cover distinct subsets of ASD would parallel the concept of core symptoms in ASD, based on the hypothesis that a group of protein factors converges on common pathways to be targeted.

Despite the translational issues in modeling ASD in animals—extreme phenotypic variability, lack of a biomarker, and appropriate endpoints for evaluation of changes in social behavior—animal models have made major contributions that have allowed the translation of basic research to clinical testing. The next generation of animal models carrying human mutations will be of the utmost importance in uncovering the neural and molecular bases of E/I imbalance and paving the way to clinical trials.

### Future Directions

Novel modulators of E-I imbalance are under study, and currently there is a modified form of memantine—nitrosynapsin—that holds promise for this purpose. Nytrosynapsin has been tested in preclinical models of ASD and demonstrated a neuroprotective action that attenuates E/I imbalance and has the potential of modifying the core defects of ASD (Ghatak et al., 2021). In addition, oxytocin might be a promising E/I modulator agent for social impairment as the oxytocinergic system is related to GABA-mediated E/I control within the dynamic interplay of social interaction. The involvement of OXT includes both endogenous and exogenous release in a still poorly known mechanism (Lopatina et al., 2018).

However, the central role of OXT in the social brain has been further demonstrated, and future studies are anticipated to evaluate the novel modifiers of E/I imbalance in ASD.

Transcranial magnetic stimulation (TMS) can be applied as a therapeutic modality in ASD to restore the imbalance of excitation and inhibition, and modulate synaptic plasticity and gamma oscillations (Oberman et al., 2016; Uzunova et al., 2016; Khaleghi et al., 2020; Casanova et al., 2021). A recent meta-analysis showed a moderate though significant effect of TMS on repetitive behaviors and core social dysfunction in ASD (Barahona-Corrêa et al., 2018). It is worth noting that TMS may be preferred in patients who cannot take medications or are not responsive to therapies that may be combined with medications, but further studies are needed.

### Limitations

The lack of preclinical data from animal models in this study is a major drawback and would have been informative, but this was out of scope since we focused on current clinical trials in ASD. Another limitation that needs to be mentioned is that the selection of the studies was based on subjective criteria based on availability of clinical trials in ASD according to our criteria that are overall in low number and need further studies.

## Author Contributions

RC designed the study and wrote the manuscript. RP contributed to revise the manuscript and customized the table. Both authors contributed to the article and approved the submitted version.

## Conflict of Interest

The authors declare that the research was conducted in the absence of any commercial or financial relationships that could be construed as a potential conflict of interest.

## Publisher’s Note

All claims expressed in this article are solely those of the authors and do not necessarily represent those of their affiliated organizations, or those of the publisher, the editors and the reviewers. Any product that may be evaluated in this article, or claim that may be made by its manufacturer, is not guaranteed or endorsed by the publisher.
